# Dietary Supplementation of Lysophospholipids Affects Feed Digestion in Lambs

**DOI:** 10.3390/ani9100805

**Published:** 2019-10-15

**Authors:** Qin Huo, Bo Li, Long Cheng, Tingting Wu, Peihua You, Shuanghua Shen, Yiyong Li, Yuhua He, Wannian Tian, Rongquan Li, Changsheng Li, Jianping Li, Baijun Song, Chunqing Wang, Xuezhao Sun

**Affiliations:** 1 The Innovation Centre of Ruminant Precision Nutrition and Smart and Ecological Farming, Jilin Agricultural Science and Technology University, Jilin 132109, China; huoqin0605@163.com (Q.H.); ma_lee@163.com (B.L.); ttw528@126.com (T.W.); yhyttt0235@sina.com (Y.H.); wannian2000@hotmail.com (W.T.); 16643429184@sohu.com (R.L.); csli-64@163.com (C.L.); sxljp2004@126.com (J.L.); jlsbj2005@126.com (B.S.); chunqingw@sina.com (C.W.); 2 Faculty of Veterinary and Agricultural Sciences, Dookie Campus, The University of Melbourne, Victoria 3647, Australia; long.cheng@unimelb.edu.au; 3 Portal Agri-Industries Co., Ltd., Nanjing 211803, China; ph-u@163.com (P.Y.); liyiyongfeed@163.com (Y.L.); 4 Floor 15, Building B, 650 Xinzhuang Avenue, Xinqiao Town, Songjiang District, Shanghai 201612, China; sshqz@sina.com

**Keywords:** lysophospholipid, growth performance, digestibility, rumen function, blood metabolite, sheep, pelleted complete feed

## Abstract

**Simple Summary:**

Previous works showed that supplementation of lysophospholipid as a feed additive improves animal growth and milk yield in beef and dairy cattle production. However, its effects on fattening lambs have not been reported before. In this study, we fed fattening lambs a diet with no or 0.5 g lysophospholipid in a kilogram of diet. We found that lysophospholipid did not or slightly improved the growth of fattening lambs. Feed digestibility, ruminal fermentation parameters and rumen bacterial community were altered, which may be associated with decreased fiber digestion. However, lipase concentration in serum was decreased, which might enhance fat deposition in muscle and thus may increase meat quality. Effects of lysophospholipid on sheep observed in this study are different from those on cattle, which warrants further study.

**Abstract:**

Five experiments were conducted to examine effects of lysophospholipids (LPL) on live weight gain, nutrient digestibility, ruminal fermentation parameters, serum biochemical parameters and rumen bacterial community profile in fattening lambs. Two dietary treatments (pelleted complete feed supplemented without (control diet; CON) or with 0.05% LPL on dry matter basis) were tested in these experiments. Feed and water were provided *ad libitum* to lambs. The results showed that average daily gain (ADG) tended to increase or was not affected by LPL supplementation. Compared with CON, the supplementation of LPL resulted in an increase in dry matter, crude protein and organic matter digestibilities, and a decrease in neutral detergent fiber and acid detergent fiber digestibilities. Ruminal pH values did not change with LPL supplementation, but the concentrations of ammonia and total short chain fatty acids (SCFAs) were increased. The molar proportion of major individual SCFAs and the ratio of acetate to propionate were not affected by LPL supplementation. While the activity of lipase was decreased with LPL supplementation, all other serum biochemical parameters did not change. Rumen bacterial community was altered by LPL supplementation with the relative abundance of fibrolytic bacteria in the total bacterial population, such as *Prevotella*, decreased. In conclusion, LPL supplementation can alter feed digestion, but may not result in consistent positive responses in animal growth performance.

## 1. Introduction

Pelleted complete feed has been increasingly applied to sheep farming in recent years [1,2]. The indoor sheep production system based on pelleted complete feed provides us with more opportunities than the grazing system, as it allows better manipulation of rumen fermentation, as well as increased feed efficiency and animal growth performance. A wide range of feed additives has been studied and applied for rumen fermentation manipulation in sheep production [3,4,5,6]. In addition to directly providing nutrients, such as rumen-protected lysine, the supplementation of microbial additives and antibiotics has been the major nutritional approach to altering rumen function and animal performance [7,8]. However, microbial additives are normally unstable in their quality and consequently in the response of animal performance [7]. Antibiotics, such as monesin, have been banned in some countries due to concerns over the formation of antibiotic-resistant microbial mutants [8,9]. This leads animal scientists to keep seeking new feed additives to increase animal production. 

Lysophospholipids (LPL) are produced from the enzymatic hydrolysis of phospholipid with a chain of fatty acids removed, mainly containing lysophosphatidylcholine, lysophosphatidic acid, lysophosphatidylethanolamine, and lysophosphatidylinositol [10]. The LPL can selectively inhibit the growth of Gram-positive but not Gram-negative bacteria [11]. They also could emulsify dietary fat [12] and upregulate genes in the intestinal epithelium [13] to increase fat absorption. This group of compounds has been used as feed additives in pig and poultry for improved feed efficiency and increased growth rate [14,15,16,17]. 

To our best knowledge, limited study has been conducted with LPL supplementation to sheep, though a few trials were performed on dairy cows [18,19,20] and beef cattle [21]. Lee et al. [19] found that adding 0.05−0.075% LPL (dry matter (DM) basis) to the diet of lactating dairy cows can increase milk production. Our preliminary trials showed that adding 0.5 mg LPL/kg DM to the diet for lactating cows increased milk yield from 28.0 to 29.5 L/day/cow. The positive animal performance might result from the emulsifying effect of LPL as emulsifying agents could increase enzymatic activities in the rumen [22] and also from the selective inhibition of Gram-positive bacteria, such as *Roseburia* spp. in the rumen [11]. 

The hybrid of thin-tailed sheep and Northeast fine-wool sheep was the most commonly farmed breed in Northeast China [23]. This breed has a relatively high growth performance and is well accommodated to the local environment. 

We hypothesized that the addition of LPL to pelleted formulated complete feed for fattening lambs may result in more efficient feed digestion and rumen fermentation towards more propionate and less acetate formation, which consequently may lead to more nutrients available for growth. This study aimed to investigate the effect of the addition of LPL to the pelleted diet of fattening lambs on animal growth performance, blood parameters, feed digestibility, ruminal fermentation parameters and rumen bacterial community.

## 2. Materials and Methods 

The use of animals and all animal handling and management in this study were approved in advance by the Committee of Animal Ethics and Welfare of Jilin Agricultural Science and Technology University (Approval number 2018001).

### 2.1. Experimental Design and Animals

The study included two dietary treatments: a control diet (CON) and a control diet supplemented with 0.5 g/kg DM of LPL. It was conducted in five experiments at three experimental sites. Experiment 1 (Exp 1) was for the determination of growth performance, digestibility, blood parameters, ruminal fermentation parameters and rumen bacterial community, while the other experiments (Exps 2–5) were for the determination of growth performance only.

Healthy hybrids of thin-tailed sheep and Northeast fine-wool sheep with similar liveweight were used for the study. All sheep were negative in the rose bengal plate test for brucellosis (*Bruceila* spp.) [24].The slow-growing sheep with daily liveweight gain less than 100 g/day during the adaptation period were excluded from the study. Sheep were stratified by liveweights measured at the end of the adaptation period and randomly allocated to one of the two dietary treatments. During the formal experimental period, sick lambs or pregnant female lambs were excluded from the study.

Exp 1 was conducted at the Animal Experimental Station of Jilin Agricultural Science and Technology University, Jilin city, Jilin province, China. Eighteen three-month-old ram lambs with a liveweight of 24.7 ± 1.9 (mean ± SD) kg were adapted for 10 days and then 14 of them with the best growth performance were randomly allocated to one of two groups with eight lambs each. After three more days for further adaptation, the formal experimental period started and lasted for 68 days (from 19 August to 26 October 2018). Sheep were fed in a half-opened feedlot and transferred to individual metabolic cages on day 15 (3 September 2018) for digestibility measurements. They were adapted to the cage housing conditions for 4 days and then the digestibility sample collection period lasted for 7 days. After the digestibility trial, animals were transferred back to the feedlot. Rumen samples were taken on days 29 and 68 (17 September and 26 October 2018) and blood samples were taken on day 42 (30 September 2018).

Exp 2 had the same experimental design as the Exp 1 and was conducted at the same site as the Exp 1, but the ram lambs were heavier and older than those in the Exp 1. The initial liveweight was at 43.8 ± 4.0 kg and aged at five months. After two weeks of isolation for the detection of brucellosis, 16 lambs were allocated to one of the two dietary treatments with seven each, housed in a pen for each treatment, and adapted to their assigned diets. The formal experimental period lasted for 28 d (from 27 September to 25 October 2018).

Exp 3 was conducted at Jiaogeermiao sheep farm in Tongyu county, Jilin province, China. Thirty six ewe lambs with a liveweight of 25.0 ± 3.1 kg were adapted to pelleted complete feed for 10 days from 11 to 21 August 2018 and then assigned to one of the two treatment groups. The formal experimental period lasted for 29 days and ended on 19 September 2018. 

Exp 4 and Exp 5 were conducted at the Portal Chifeng Experimental Station, Inner Mongolia, China. Exp 4 had ram lambs with a liveweight of 29.9 ± 5.4 kg and in Exp 5 ewe lambs had a liveweight of 27.0 ± 3.5 kg. Lambs were adapted to pelleted complete feed for 14 days and then were weighed and grouped by weight. Lambs from each treatment were fed in two pens with the equal number of animals. The formal experimental period lasted for 62 days from 7 November 2018 to 8 January 2019. Animals were weighed on days 0, 24 and 62. At the end of the experiment, all lambs were slaughtered and hot carcasses were weighed.

### 2.2. Diets and Feeding Management

The experimental diets were formulated according to the Chinese Feeding Standard for Lamb Finishing (NY/T 816-2004). The ingredients and nutrient contents of the diets are listed in Table 1. Sorghum husk was ground using a 2 mm sieve and maize, maize germ meal, sunflower seed meal, peanut shell, rice hull, cottonseed meal, and barley malt root using a 4/6 mm sieve (half sieve with holes having a diameter of 4 mm and another half 6 mm).

All ingredients were thoroughly mixed, conditioned at 90 °C for 45 s and pelletized at 85 °C using a pelleting machine with the compression ratio of ring to die at 1:7 (model YPM508E; Jiangsu Yongli Machinery Co., Ltd., Liyang City, Jiangsu Province, China). The pellets were air-cooled. Feed pellets were 5 mm in diameter and 8−10 mm in length. All feed used in the whole period of the experiments was pelleted in one batch at the Chifeng Subsidiary Company of Jiangsu Portal Agri-Industries Co., Ltd.

During the adaptation period, feed provided to lambs was gradually changed from hay to pellets by an increase of 100 g of pellets a day. Pellets provided in the first week contained an antiparasitic agent (triclabendazole, 250 mg/kg feed) and then experimental pellets were provided. Feed was provided twice a day with equal portions at 8:00 and 17:00. Once animals were completely on pelleted feed, feed allowance was set to allow leftover at ca. 10% of total feed provided on the previous day to achieve feeding *ad libitum*. Leftovers were cleaned up and weighed before morning feeding for intake calculation. Drinking water was available all the time. Weather, temperature and humidity were recorded daily, and animal behaviors were observed.

### 2.3. Liveweight Measurement and Sampling

Liveweight was measured before morning feeding after 16 h fasting with an accuracy of 0.05 kg using an electronic scale for living bodies (YongHuang, Jinhua, Zhejiang, China) at the beginning and the end of the formal experimental period, and in the middle of the experiment if the experimental period was longer than one month. Average daily gain (ADG) was calculated as the slope of liveweight against date.

Rumen sample (50 mL) was taken from each lamb in the Exp 1 via mouth using stomach tubing (1500 mm in length, 8 mm in inner diameter, 6 openings with a diameter of 2 mm in the end to the rumen, made with polyurethane material) at 0 and 3 h after morning feeding. Ruminal pH value was measured immediately after sampling using a pH meter with the accuracy of 0.01 (LICHEN pH-100A, Shanghai Lichen Scientific Laboratory Instrument Ltd., Shanghai, China). The rumen sample was placed in a 2-mL cryogenic vial with the circular bottom (Corning Inc., New York, USA) and then stored at −20 °C for the analysis of short chain fatty acid (SCFA) and ammonia concentrations. The remaining samples (3 h after morning feeding samples only) were stored at −80 °C for the characterization of rumen microbial profile. 

Blood sample (5 mL) was collected into a coagulation promoting tube with separating gel (Sanli Industrial Co., Ltd., Huizhou, China) from the jugular vein of each lamb in the Exp 1 before morning feeding.

### 2.4. Digestibility Trial

Digestibility measurement was conducted in metabolic cages using the total feces collection technique. The digestibility trial lasted for 10 days, including a 3-day adaptation and 7-day collection periods. Feed allowance was adjusted to allow 5−10% refused and feed provided at 8:00 and 15:00 with equal portions. Feed sample (50 g) was collected daily. Refusals, feces and urine were collected and quantified daily. Refusals were all kept and feces and urine subsampled. One fifth of collected feces was stored at −20 °C, with half acidified with 10% H_2_SO_4_ at a ratio of 1:10 for crude protein (CP) determination and another half for the determination of other nutrients. Total urine was collected in a tank with 100 mL of 10% H_2_SO_4_ added and diluted to 3000 mL, from which 30 mL was taken and kept at −20 °C. At the end of the experiment, samples were pooled over individual animals. Feed, refusal and feces samples were dried at 65 °C for 48 h and stored in cold for later analysis. Data from one sheep in the LPL group were discarded due to health problem of the sheep.

### 2.5. Laboratory Analysis

Feed samples were analyzed for DM, organic matter (OM), CP, neutral detergent fiber (NDF), acid detergent fiber (ADF), ether extract (EE), Ca and P, refusal and feces samples for DM, OM, CP, NDF and ADF, and urine samples for CP. Crude protein was determined in the modified method of Kjeldahl (method GB/T 6432-2018) using an automatic nitrogen determination apparatus (model K9860; Haineng Instruments Ltd., Jinan, Shandong, China). Dry matter was determined by the loss of water during drying at 105 °C for 3 h (method GB/T 6435-2014). Ash was determined by samples heated until no smoking and then in a muffle at 550 °C for 4 h (GB/T 6438-2007). Ether extract was determined as dry matter loss after extraction in petroleum ether for 24 h at 30–60 °C (GB/T 6433-2006). NDF and ADF were consecutively determined after boiling in 3% neutral detergent for 1 h (GB/T 20806-2006) and 2% acid detergent for 1 h (NY/T 1459-2007), respectively.

A cryogenic vial with a rumen sample was thawed in tap water and centrifuged at 2000× *g* for 10 min. The supernatant was transferred to a 5 mL centrifugation tube, mixed with 25% metaphosphoric acid at a 4:1 ratio, vortexed and centrifuged at 4 °C in 8000× *g* for 10 min. The supernatant from this centrifugation was filtered by a 0.22 μm filter for water solution (Shanghai Rebus Biotechnology Co., Ltd., Shanghai, China) and transferred to a new 5 mL centrifugal tube for the determination of SCFA and ammonia concentrations. 

Short chain fatty acids were identified and quantified using a gas chromatograph system (GC9790, Fuli Instruments Ltd., Wenling, Zhenjiang, China) fitted with a flame ionisation detector (FID). The GC was fitted with a FFAP 30 m × 3 mm × 0.25 μm capillary polar column. The column temperature was set at 140 °C, the FID set at 250 °C and vaporization chamber temperature at 250 °C with N_2_ as a carrier gas and shunt ratio at 1:50. The injection volume was 1 μL.

The above filtered rumen sample was diluted with double evaporated water in a ratio of 1:7 and vortexed in a 5 mL centrifugal tube. Ammonia concentration was determined using the Indigo phenol blue-spectrophotometry method [26] modified by Feng and Gao [27]. Briefly, the sample solution was mixed with a color developer 37 °C for 20 min, cooled down with tap water to room temperature, and measured for absorbance at 637 nm.

The collected blood sample was centrifuged at 1000× *g* for 5 min (Model TDL-80-2B; Anting Scientific Instrument Factory, Shanghai, China) and the serum analyzed for blood biochemical parameters using an automatic biochemical analyzer (Model 7160; Hitachi Ltd., Tokyo, Japan). The reagents for the analysis were purchased from Mairui Biomedical Electronics Co., Ltd. (Shenzhen, China).

Rumen bacterial populations were analyzed at Shanghai Biozeron Biotechnology Co., Ltd. (Shanghai, China). Total genome DNA was extracted using the CTAB/SDS method, monitored for DNA concentration and purity on 1% agarose gels and then diluted to 1 ng/μL. The 16S rRNA genes were amplified using hypervariable V3-V4 region PCR primers (341F: 5′- CCTAYGGGRBGCASCAG-3′; 806R: 5′- GGACTACNNGGGTATCTAAT-3′) with the barcode. All PCR reactions were carried out in 30 μL reactions with 15 μL of Phusion^®^High-Fidelity PCR Master Mix (New England Biolabs, Ipswich, MA, USA), 0.2 μM of forward and reverse primers and about 10 ng template DNA. Thermal cycling consisted of initial denaturation at 98 °C for 1 min, followed by 30 cycles of denaturation at 98 °C for 10 s, annealing at 50 °C for 30 s, elongation at 72 °C for 60 s and finalization at 72 °C for 5 min. After amplification, PCR products were mixed with the same volume of SYBR green (QIAGEN Inc., Alameda, CA, USA) containing buffer and then operated electrophoresis on 2% agarose gel. Samples with bright main strip between 400–450 bp were purified with GeneJET Gel Extraction Kit (Thermo Fisher Scientific (China) Ltd., Shanghai, China). Sequencing libraries were generated using NEB Next^®^Ultra™DNA Library Prep Kit for Illumina (New England Biolabs, Ipswich, MA, USA) following the manufacturer’s recommendations. The library quality was assessed on the Qubit^@^ 2.0 Fluorometer (Thermo Fisher Scientific (China) Ltd., Shanghai, China) and Agilent Bioanalyzer 2100 system (Agilent Technologies, Inc., Santa Clara, CA, USA). At last, the library was sequenced on an Illumina MiSeq platform (Illumina, Inc., San Diego, CA, USA) and 250 bp/300 bp paired-end reads were generated. Paired-end reads from the original DNA fragments were merged using FLASH [28], which was designed to merge paired-end reads when at least some of the reads overlap the read generated from the opposite end of the same DNA fragment. Sequences analysis were performed by UPARSE software package using the UPARSE-OTU and UPARSE-OTU ref algorithms [29]. In-house Perl scripts were used to analyze alpha (within samples) and beta (among samples) diversity. Sequences with ≥97% similarity were assigned to the same OTUs and the RDP classifier was used to annotate taxonomic information for the representative sequence in each OTU according to the database Silva (www.arb-silva.de) updated for the taxonomic assignment of ruminal bacteria [30]. Details in bioinformatics analysis are described in Appendix A.

### 2.6. Statistical Analysis

The statistical analysis was conducted using GenStat 19th edition (VSN International, Hemel Hempstead, UK, 2017) and significance was declared at *p* < 0.05. The data of liveweight, ADG, carcass weight, dressing percentage, feed intake, digestibility and blood biochemical parameters were analyzed with a one-way ANOVA. Rumen fermentation parameters were analyzed using a mixed model (REML) with repeated measurements. Treatment, sampling time and the interaction of treatment and sampling time were defined to have fixed effects in the model, sampling date, and animal as random effects. Parameters obtained from each lamb at different sampling times were treated as repeated measures. A meta-analysis was conducted for ADG with experimental site using block and dietary treatment, sex, and their interaction as fixed effects. As the interaction was not significant, it was omitted from the model for analysis. For the data of ruminal bacterial community, only when average relative abundance of a species in any treatment group was over 0.5%, the species and its corresponding genus and phylum were statistically analyzed with a one-way ANOVA. 

## 3. Results

### 3.1. Growth Performance

In Exp 1, the initial liveweight was similar for the two treatments (*p* = 0.826), but after 68 days the final liveweight tended to be higher for the LPL treatment than for the control (*p* = 0.084; Table 2). The LPL treatment was 12% higher in ADG than the control, however, the difference did not reach the level of significance (*p* = 0.165). In Exp 2, the supplementation of LPL tended to increase ADG (*p* = 0.099). In other experiments, a significant change in ADG with LPL supplementation was not observed (*p* ≥ 0.601). The meta-analysis with all ADG data obtained in this study showed that the supplementation of LPL (*p* = 0.471) or the interaction of LPL and animal sex (*p* = 0.400) did not significantly change ADG, although male lambs (274 g/day) had higher (*p* < 0.001) ADG than female lambs (200 g/day).

At the end of Exp 3 and Exp 4, all lambs were slaughtered. The supplementation of LPL did not significantly (*p* ≥ 0.276) affect hot carcass weights for either male or female lambs. Dressing percentage was similar for the two treatments in female lambs. However, male lambs had a lower dressing percentage (*p* = 0.012) by 1.4 unit when the diet was supplemented with LPL than with the control.

### 3.2. Total Tract Nutrient Apparent Digestibility

During the period of digestibility trial, dry matter intake and other nutrient intakes were similar for the treatment and control animals (*p* ≥ 0.363; Table 3). The supplementation of LPL slightly (by 2%), but statistically significantly (*p* < 0.05) increased DM and OM digestibilities. The increase in CP digestibility resulted from the supplementation of LPL was 4.3% (*p* = 0.024). However, NDF and ADF digestibilities had both statistically (*p* = 0.012 for NDF and *p* = 0.001 for ADF, respectively) and biologically (by −8% for NDF and −35% for ADF, respectively) significant decreases. The digestibility of EE did not significantly (*p* = 0.263) change with the supplementation of LPL.

### 3.3. Rumen Fermentation Parameters

Although ruminal pH value was higher before morning feeding than 3 h after morning feeding (*p* < 0.001), the supplementation of LPL did not significantly affect ruminal pH value (*p* = 0.235; Table 4). Both LPL supplementation (*p* = 0.011) and sampling time (*p* < 0.001) significantly affected ammonia-N concentration in the rumen and there was no significant interaction between them. The supplementation of LPL resulted in 37% higher ammonia-N concentration compared with the control. Supplementation, sampling time and their interaction significantly affected or tended to affect total SCFA concentration (*p* ≤ 0.083). Although the total SCFA concentration at 3 h after morning feeding was similar for both LPL supplemented and unsupplemented lambs, the supplementation of LPL increased the total SCFA concentration before morning feeding by 61% (*p* < 0.05). In term of the molar proportion of individual SCFAs and the ratio of acetate to propionate, LPL supplementation and its interaction with sampling time did not have significant effects (*p* ≥ 0.172) although sampling time did significantly affect the molar proportions of acetate and propionate and their ratio (*p* ≤ 0.026).

### 3.4. Blood Metabolites

Among serum biochemical parameters measured in this study (Table 5), only lipase activity was significantly (*p* = 0.034) altered by the supplementation of LPL and the activity of the enzyme decreased by 71% compared with the control.

### 3.5. Rumen Bacterial Community

**Figure 1:**
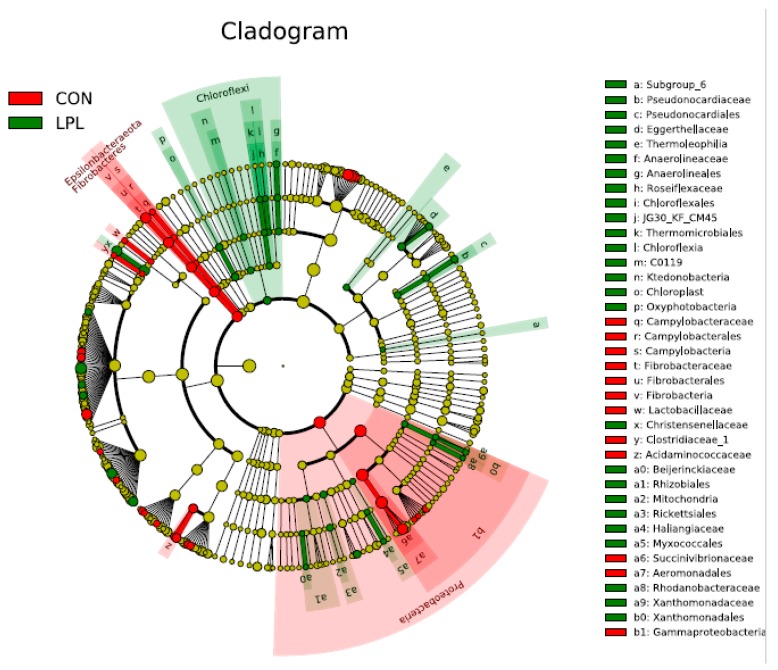
LEfSe cladogram of the composition of bacterial community in the rumen fattening lambs fed a diet supplemented without (CON) or with (LPL) 0.05% lysophospholipid. Differences are represented in the color of the group, where taxa are most abundant. Red: taxa abundant in CON; Green: taxa abundant in LPL.

**Figure 2:**
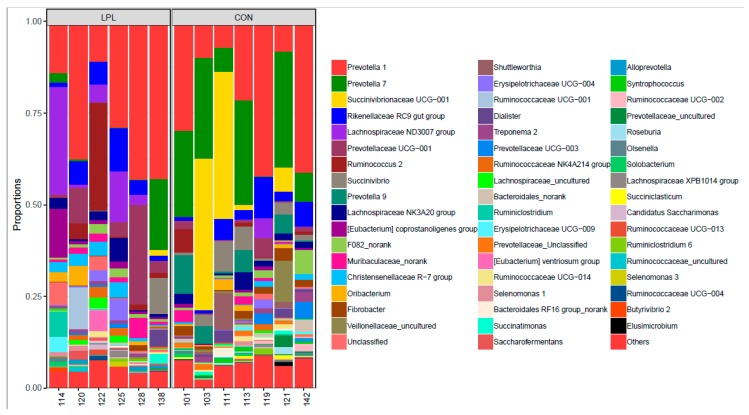
Distribution of the most dominant genera in the rumen of fattening lambs fed a diet supplemented without (CON) or with (LPL) 0.05% lysophospholipid (those with a relative abundance of less than 1% were combined as other).

The supplementation of LPL altered the composition of bacterial community in the rumen (Figure 1). At the phylum level, the dominant phyla were *Bacteroidetes*, *Firmicutes,* and *Proteobacteria* in the rumen of lambs fed a diet containing either LPL or no LPL, accounting for 95.4% of total bacterial relative abundance (Figure S1). The relative abundance of these three phyla were 51.5%, 41.0%, and 2.8% in LPL treatment, 58.3%, 20.0%, and 17.1%, respectively. *Firmicutes* (CON 20.0% vs. LPL 41.0%; *p* = 0.055) and *Fibrobacteres* (CON 1.87% vs. LPL 0.36%; *p* = 0.011) were significantly different between the two treatments (Figure S1; Table S1). On the average the relative abundance of *Proteobacteria* dropped over 83.5% when LPL was supplemented, although the difference was insignificant due to a large variation within treatment groups. At the genus level (Figure 2), *Christensenellaceae* (*p* = 0.012), *Lachnospiraceae* (*p* = 0.090), and *Saccharofermentans* (*p* = 0.058) were increased and *Fibrobacter* (*p* = 0.011), *Prevotella* 7 (*p* = 0.037), *Prevotella* 9 (*p* = 0.031), unclassified *Prevotellaceae* (*p* = 0.011), and *Syntrophococcus* (*p* = 0.050) were decreased with the supplementation of LPL (Supplementary Table S1). At the species level, the *Christensenellaceae* R-7 group increased from 0.6% to 2.4% (*p* = 0.012), *Erysipelotrichaceae* from 0.4% to 2.2% (*p* = 0.078), *Lachnospiraceae* from 2.7% to 11.9% (*p* = 0.089), and uncultured *Oribacterium* from 0% to 1.5% (*p* = 0.088) when LPL was supplemented, while *Fibrobacter* was decreased from 1.8% to 0.3% (*p* = 0.013) and uncultured *Syntrophococcus* from 0.5% to 0.1% (*p* = 0.036). The supplementation of LPL reduced the relative abundance of Gram-positive bacteria *Roseburia* spp. 0.11% to 0.02%.

## 4. Discussion

### 4.1. Animal Performance

In this study, the response of lambs to the supplementation of LPL at 0.05% of dietary DM was inconsistent. The ADG was increased in two experiments, but the difference did not reach the significance level (Exp 1) or just reached the tendency level (Exp 2). In the other three experiments, ADG did not change with the supplementation of LPL. There are no reported studies with LPL supplemented to sheep diet in the literature for us to directly compare with. In other ruminant species, the response of animal production to LPL supplementation was inconsistent as well. Song et al. [21] found that the supplementation of LPL at 0.3−0.5% of dietary DM to the diet of beef cattle did not improve growth performance. However, our preliminary trial showed that eleven 15.5-month-old Hanwoo beef cattle supplemented with LPL at 0.1% of the diet for 3 months had an average weight gain of 123.4 kg, being higher than the average weight gain of 117.5 kg from the control animals. Rico et al. [18] supplemented LPL at 10 g/day (approximately 0.035% of dietary DM) to lactating dairy cows for 10 days and did not observe an increase of milk yield. In contrast, a short-term supplementation of LPL at 0.05−0.075% of dietary DM did increase milk yield by 5.5%−6.5% [19].

The inconsistency in animal production in ruminants is contradictory to the studies on non-ruminants, in which LPL consistently increased growth and lactation performance and feed efficiency [14,15,16]. The inconsistent results among different ruminant studies might result from the variation in source of LPL (Sontakke et al. [20] vs. Lee et al. [19]), the dose of LPL (this study vs. Song et al. [21]), the duration of supplementation (e.g., this study vs. Lee et al. [19]), active compounds in LPL, the degradation of LPL in the rumen, etc. The degree of the removal of fatty acid chain from phospholipid during enzymatic hydrolysis affects the bioactivity of LPL [31]. The studies conducted previously might have used LPL with lower activity than the recent studies, which is expected to have different responses. It is well known that feed is subjected to degradation in the rumen before further digestion in a way similar to nonruminants. Phospholipids, from which LPL is produced, can be degraded in the rumen, but a considerable amount of LPL was able to bypass the rumen [32]. The degree of degradation may vary with the source of LPL and rumen environment which is moderated by diets and other factors [33]. We are not sure how much LPL can escape from the rumen. The discrepancy in growth response among experiments within this study may result from the age of animals, seasons and environment which all might affect rumen condition for degradation. In the future, studies are needed to determine the degradation of LPL in the rumen and to explore rumen-protected LPL.

### 4.2. Digestibility

To the best of our knowledge, there are only two studies in the literature measuring digestibility when LPL is supplemented to a diet for ruminants (Song et al. [21] in beef cattle and Lee et al. [19] in dairy cows). We found that LPL supplementation increased DM digestibility, which is consistent with the findings of Song et al. [21] who supplemented LPL to beef cattle diet at 0.3%–0.5% of dietary DM, but is contrasted with the results obtained from lactating dairy cows where DM digestibility slightly decreased when LPL was supplemented at 0.05−0.075% in the diet [19]. Although LPL supplementation resulted in an increase (this study) or a decrease [19] in DM digestibility, the difference between LPL and CON was only 12−14 g/kg. This small difference may not be biologically significant. However, in the study by Song et al. [21], the difference was small, only 8−17 g/kg on day 30, but increased with time. After 90 days, the difference in DM digestibility increased by 64 g/kg for LPL supplementation at 0.5% of the diet. Our digestibility trial started on day 15 of the experimental period. The measurement should be taken after LPL is supplemented for a longer time as well for better understanding in the effect of LPL on digestibility.

Song et al. [21] measured DM digestibility only. Lee et al. [19] is only a reference for comparison in nutrient digestibility. In our study, OM digestibility was increased, but decreased in the study conducted by Lee et al. [19]. Being similar to DM digestibility, the difference between LPL and CON was small as well and might be not relevant biologically at least for a short-term after LPL supplementation.

Our result showed that CP digestibility was increased, while it did not change in the study by Lee et al. [19]. The increase in CP digestibility was consistent with increased ruminal ammonia-N concentration 3 h after morning feeding with LPL supplementation. How LPL affect CP digestion is not known and warrants further study.

NDF digestibility decreased by around 40 g/kg DM in both Lee et al. [19] and our study, although the decrease was not statistically significant in the study by Lee et al. [19]. Lee et al. [19] did not measure ADF digestibility. Our result showed that LPL supplementation resulted in a large decrease in ADF digestibility by 99 g/kg DM. The great decrease in fiber digestibility is conflicted with our hypothesis proposed from the finding that fiber degradation is improved by emulsifying agents due to increased cellulolytic enzyme activity [34]. Further study is warranted for the mechanisms underlying the inhibition of fiber degradation by LPL.

### 4.3. Rumen Fermentation Parameters

Our study was contrasted with Lee et al. [19]’s findings on rumen fermentation parameters although ruminal pH value was not affected by LPL in both studies. We found ammonia and total SCFA concentrations were increased with LPL, but Lee et al. [19] did not. There was a lack of difference in the molar proportion of major individual SCFAs and the ratio of acetate to propionate in both studies. The reason for different effects of LPL on rumen fermentation is not known, but the chemical composition of the diet, especially EE content, was greatly different between the two studies, which might alter LPL reaction in the rumen. Our results did support the finding that LPL enhances feed digestion, but did not support the hypothesis that LPL results in rumen fermentation towards more propionate and less acetate formation that we proposed before.

### 4.4. Blood Biochemical Parameters

The major change observed in blood biochemical parameters was a decrease in lipase activity associated with the supplementation of LPL. Rico et al. [18] found that milk fat concentration was increased 5 days after dairy cows were supplemented to a higher fiber and lower unsaturated fatty acid diet. These authors attributed the increase in milk fat concentration to increased acetate supply. We suspect their findings might be associated with the decrease in lipase activity. How LPL affects lipase activity warrants further mechanistic investigation. We suspect LPL that escaped from the rumen is absorbed in the small intestines and stimulates the body to depress the expression of genes encoding lipase. From another point of view, the decrease in lipase activity might enhance fat deposition in muscle, which could be useful for the production of quality meat. 

Most blood biochemical parameters tested did not alter with the supplementation of LPL, suggesting the status of energy and nitrogen in the body has not been improved, which supports the outcome of no or a slight difference in ADG between the two treatment groups.

### 4.5. Rumen Bacterial Community Profile

A significant change in bacterial community profile was observed when LPL was supplemented to the diet for lambs. Of the most abundant genera found in the study, *Prevotella* was most significantly decreased with LPL supplementation based on LDA effect size factors. *Prevotella* is well-known hemicellulose degrading bacteria [35]. The decreased abundance of *Prevotella* was consistent with our finding that fiber digestibility was decreased with LPL. It is worthy to mention that among the *Prevotella* bacteria, *Prevotella* 1 was increased, while *Prevotella* 7 and *Prevotella* 9 decreased with LPL, suggesting a wide diversity of *Prevotella* bacteria in their biochemical characteristics.

*Lachnospiraceae* ND3007 group was increased, having the largest LDA effect size factor, with LPL supplementation in the diet. The bacteria in this genus was found to be decreased with an increase in temperature and humidity index [36]. The genus has not been well described in the literature, but is a butyric acid producing strain and considered as a probiotics [36]. The increase of the genus might promote a healthy rumen environment. 

To date, the six culturable lipolytic bacteria are *Anaerovibrio lipolytica* and some species in the genera *Butyrivibrio*, *Clostridium,* and *Propionibacterium* [37]. The populations of these bacteria were quite small in the rumen of lambs fed the two diets in this study. This reflected the low content of ether extract (1.6 g/kg) in the diet. The content is at the low end of the lipid content range of 20 to 100 g/kg in the diet for ruminants [38]. The abundance of these bacteria was not changed with LPL supplementation, suggesting lipid metabolism in the rumen is not affected. The decreased activity of lipase in serum in the lambs fed a diet supplemented with LPL might just result from the absorption of LPL into the blood and was not related to lipid metabolism in the rumen.

*Roseburia* spp., Gram-positive bacteria, can be selectively inhibited by LPL in vitro [11]. This is confirmed true in vivo as the relative abundance of the genus in the rumen dropped from 0.11% to 0.02% with the supplementation of LPL in this study although the genus might be not biologically important for fattening lambs due to the low abundance. However, the phylum *Firmicutes* belongs to Gram-positive bacteria, increasing from 20% to 41% in the relative abundance. It might be because LPL may not inhibit all Gram-positive bacteria or the LPL concentration is not high enough to have an inhibitory effect.

## 5. Conclusions

The supplementation of lysophospholipid did not consistently result in positive animal performance in fattening lambs in this study. Further studies on a large scale are warranted to confirm the effect of lysophospholipid in animal performance. Feed digestion and rumen fermentation were altered with lysophospholipid supplementation, which was in the opposite direction to the results reported in other ruminant species and contradictory with our hypothesis that lysophospholipid enhances fiber degradation. Mechanism studies are needed to explore the effects of lysophospholipid on rumen fermentation, ruminal degradation and bypass of lysophospholipid, and body metabolisms, especially fat metabolism.

## Figures and Tables

**Table 1:** Ingredients and nutrient contents of experimental diets supplemented without (CON) or with (LPL) lysophospholipids.

Item	Diet	
	CON	LPL
Ingredient (kg/t of fresh weight)		
Maize	350	350
Maize germ meal	120	120
Sunflower seed meal	120	120
Peanut shell	113	113
Rice hull	70	70
Cottonseed meal	30	30
Bentonite	20	20
Barley malt root	100	100
Limestone	14	14
Sorghum husk	10	10
Calcium hydrogen phosphate	7	7
Soybean powder	20	20
Sodium chloride	6	6
Premix (Trace mineral salt and vitamins) ^1^	20	20
Lysophospholipids		0.5
Nutrient contents ^2^ (g/kg of DM)		
Dry matter (DM) (g/kg of fresh weight)	880	874
Organic matter (OM)	903	905
Crude protein (CP)	158	162
Neutral detergent fiber (NDF)	427	435
Acid detergent fiber (ADF)	174	178
Ether extract (EE)	16	16
Metabolizable energy (MJ/kg of DM) ^3^	11.7	11.8

^1^ Premix per kg contained 24,000 IU vitamin A, 4800 IU vitamin E, 120 mg Fe, and 24 mg Cu; ^2^ The nutrient contents were measured values. ^3^ Metabolizable energy was estimated from NRC (2007) [25].

**Table 2:** Growth performance of fattening lambs fed experimental diets supplemented without (CON) or with (LPL) lysophospholipids.

Experiment No.	Site	Sex	Item	Diet		*p* Value
				CON	LPL	
Exp 1	Jilin	Male	n	7	6	
			Days	68	68	
			Initial liveweight (kg)	24.6 ± 0.56	24.8 ± 0.58	0.826
			Final liveweight (kg)	44.6 ± 1.12	46.9 ± 0.86	0.084
			Average daily gain (ADG) (g/day)	294 ± 17.3	330 ± 8.1	0.165
Exp 2	Jilin	Male	n	8	8	
			Days	28	28	
			Initial liveweight (kg)	43.6 ± 1.34	44.7 ± 1.38	0.568
			Final liveweight (kg)	48.9 ± 1.65	52.4 ± 1.74	0.158
			Average daily gain (ADG) (g/day)	198 ± 38.9	282 ± 24.3	0.099
Exp 3	Tongyu	Female	n	18	17	
			Days	29	29	
			Initial liveweight (kg)	24.9 ± 0.75	25.0 ± 0.77	0.972
			Final liveweight (kg)	30.9 ± 0.93	30.7 ± 0.96	0.851
			Average daily gain (ADG) (g/day)	207 ± 13.2	197 ± 13.5	0.601
Exp 4	Chefeng	Male	n	23	26	
			Days	62	62	
			Initial liveweight (kg)	30.4 ± 1.17	31.6 ± 0.76	0.378
			Final liveweight (kg)	47.5 ± 1.56	48.5 ± 0.92	0.569
			Average daily gain (ADG) (g/day)	276 ± 12.3	275 ± 9.6	0.982
			Carcass (kg)	20.3 ± 0.64	20.2 ± 0.47	0.815
			Dressing percentage (%)	42.9 ± 0.38	41.5 ± 0.38	0.012
Exp 5	Chefeng	Female	Number of animals	18	27	
			Days	62	62	
			Initial liveweight (kg)	28.7 ± 0.76	29.8 ± 0.65	0.286
			Final liveweight (kg)	41.0 ± 1.14	42.4 ± 0.90	0.339
			Average daily gain (ADG) (g/day)	198 ± 13.7	203 ± 8.5	0.764
			Carcass (kg)	17.4 ± 0.48	18.1 ± 0.43	0.276
			Dressing percentage (%)	42.5 ± 0.38	42.8 ± 0.34	0.621

**Table 3:** Intake and total tract apparent nutrient digestibility of fattening lambs offered a diet supplemented without (CON) or with (LPL) lysophospholipids.

Index	Diet		*p* Value
	CON (n = 7)	LPL (n = 6)	
Intake (g/day)			
Dry matter intake (DMI)	1328 ± 53.2	1382 ± 97.3	0.620
Organic matter intake (OMI)	1199 ± 48.1	1256 ± 88.3	0.566
Crude protein intake (CP intake)	207 ± 8.4	224 ± 15.7	0.363
Neutral detergent fiber intake (NDF intake)	562 ± 23.1	560 ± 46.9	0.969
Acid detergent fiber intake (ADF intake)	233 ± 9.3	240 ± 18.7	0.730
Ether extract intake (EE intake)	21.2 ± 1.83	22.2 ± 2.00	0.710
Digestibility (g/kg of DM)			
Dry matter (DM)	617 ± 3.3	629 ± 4.4	0.047
Organic matter (OM)	655 ± 3.1	669 ± 4.9	0.039
Crude protein (CP)	694 ± 7.0	723 ± 9.3	0.024
Neutral detergent fiber (NDF)	503 ± 8.3	463 ± 10.3	0.012
Acid detergent fiber (ADF)	285 ± 13.6	186 ± 17.3	0.001
Ether extract (EE)	941 ± 10.8	928 ± 1.3	0.263

**Table 4:** Ruminal fermentation parameters of fattening lambs (n = 7) fed experimental diets supplemented with (LPL) or without (CON) lysophospholipids.

Index	Diet				*p* Value		
	LPL (n = 7)		CON (n = 7)				
	Before ^1^	After ^1^	Before	After	Treatment (T)	Sampling Time (S)	T × S
Ruminal pH	6.75 ± 0.127 ^a 2^	5.94 ± 0.159 ^b^	7.01 ± 0.048 ^a^	5.98 ± 0.137 ^b^	0.235	<0.001	0.372
Ammonia N (mmol/L)	8.92 ± 1.450 ^b^	13.13 ± 1.328 ^a^	6.92 ± 0.721 ^b^	9.19 ± 0.911 ^b^	0.011	0.006	0.384
Total SCFA ^3^ (mmol/L)	44.4 ± 6.93 ^b^	65.9 ± 4.97 ^a^	27.5 ± 2.28 ^c^	66.0 ± 3.86 ^a^	0.052	<0.001	0.083
Individual SCFA molar proportion (mol/100 mol)							
Acetate	60.5 ± 0.93 ^a^	58.0 ± 1.47 ^ab^	61.6 ± 1.86 ^a^	54.9 ± 1.35 ^b^	0.627	0.003	0.172
Propionate	22.3 ± 2.35 ^b^	25.6 ± 2.35 ^ab^	26.0 ± 2.63 ^a^	31.8 ± 2.40 ^a^	0.194	0.026	0.674
Butyrate	17.2 ± 1.88	16.4 ± 2.09	12.4 ± 1.37	13.3 ± 1.86	0.151	0.930	0.340
Acetate: Propionate	2.96 ± 0.322 ^a^	2.50 ± 0.262 ^ab^	2.77 ± 0.359 ^a^	1.93 ± 0.216 ^b^	0.443	0.011	0.447

^1^ Before, rumen samples taken before morning feeding; After, rumen samples taken 3 h after morning feeding; ^2^ a, b, c: Different letters on the shoulder of values in a row mean a significant difference (*p* < 0.05); ^3^ SCFA, short chain fatty acid.

**Table 5:** Serum biochemical parameters of fattening lambs fed experimental diets supplemented without (CON) or with (LPL) lysophospholipids.

Item	Diet		Normal Range	*p* Value
	CON (n = 7)	LPL (n = 7)		
α-Amylase (AMYL) (U/L)	21.7 ± 4.54	26.8 ± 4.50	1–30	0.439
Albumin (ALB) (g/L)	27.5 ± 1.31	27.2 ± 1.31	24–37	0.854
Alkaline phosphatase (ALKP) (U/L)	214 ± 18.0	186 ± 14.0	50–228	0.249
Alkaline transaminase (ALT) (U/L)	6.93 ± 0.913	8.84 ± 1.503	5–17	0.299
Aspartate transaminase (AST) (U/L)	54.4 ± 5.71	62.9 ± 8.01	40–96	0.403
Low density lipoprotein cholesterol (LDL) (mmol/L)	0.573 ± 0.0464	0.497 ± 0.0785	–	0.423
High density lipoprotein cholesterol (HDL) (mmol/L)	0.471 ± 0.0522	0.400 ± 0.0724	–	0.439
Total cholesterol (TC) (mmol/L)	1.29 ± 0.156	1.22 ± 0.095	1.14–2.12	0.725
Urea N (UREA) (mg/dL)	9.13 ± 0.861	8.66 ± 1.180	–	0.753
Creatinine (CREA) (μmol/L)	88.0 ± 18.87	53.5 ± 14.44	53–133	0.186
Triglyceride (TG) (mmol/L)	0.223 ± 0.0231	0.210 ± 0.0225	0.10–0.34	0.700
Total protein (TP) (g/L)	75.8 ± 1.60	74.5 ± 1.58	56–78	0.565
Glucose (GLU) (mmol/L)	4.10 ± 0.210	4.19 ± 0.238	2.78–4.45	0.778
Lipase (LIP) (U/L)	61.4 ± 16.29	17.8 ± 3.24	1–71	0.034
Globulin (GLOB) (g/L)	24.2 ± 1.01	25.6 ± 2.38	32–41	0.585

## Supplementary Materials

The following are available online at https://www.mdpi.com/2076-2615/9/10/805/s1, Figure S1: Distribution of the most dominant phyla in the rumen of fattening lambs fed a diet supplemented without (CON) or with (LPL) 0.05% lysophospholipid (those with a relative abundance of less than 2% were combined as other), Figure S2: Linear discriminant analysis effect size of rumen bacterial populations of fattening lambs fed a diet supplemented without (CON) or with (LPL) 0.05% lysophospholipid. This plot represents the most differentially abundant taxa according to the logarithmic linear discriminant analysis (LDA) score cutoff of at 4.0. Table S1: Ruminal bacterial composition (% of total sequence reads) in fattening lambs fed experimental diets supplemented with (LPL) or without (CON) lysophospholipids.

## Appendix A

Graphical representation of the relative abundance of bacterial diversity from phylum to species was visualized using Krona chart. Cluster analysis was preceded by principal component analysis (PCA) to reduce the dimension of the original variables using the QIIME software package [39]. QIIME calculates both weighted and unweighted unifrac distance, which are phylogenetic measures of beta diversity. We used unweighted unifrac distance for Principal Coordinate Analysis (PCoA) and Unweighted Pair Group Method with Arithmetic mean (UPGMA) Clustering.

To confirm differences in the abundances of individual taxonomy between the two groups, Metastats software was utilized. LEfSe was used for the quantitative analysis of biomarkers within different groups. This method was designed to analyze data in which the number of species is much higher than the number of samples and to provide biological class explanations to establish statistical significance, biological consistency, and effect-size estimation of predicted biomarkers. To identify differences of microbial communities between the two groups, ANOSIM [40] and MRPP (multi-response permutation procedure) [41] were performed based on the Bray-Curtis dissimilarity distance matrices.

## References

[1] Sun X., Song B., He Y., You P. (2017). A review on pelleted complete feed for sheep and goats. Mod. J. Anim. Husb. Vet. Med..

[2] Toteda F., Facciolongo A.M., Ragni M., Vicenti A. (2011). Effect of suckling type and PUFA use on productive performances, quanti-qualitative characteristics of meat and fatty acid profile in lamb. Prog. Nutr..

[3] Salem A.F.Z.M. (2013). Nutritional Strategies of Animal Feed Additives.

[4] Guo X., Cheng L., Liu J., Zhang S., Sun X., Al-Marashdeh O. (2019). Effects of licorice extract supplementation on feed intake, digestion, rumen function, blood indices and live weight gain of Karakul sheep. Animals.

[5] Zhong R., Xiang H., Cheng L., Zhao C., Wang F., Zhao X., Fang Y. (2019). Effects of feeding garlic powder on growth performance, rumen fermentation, and the health status of lambs infected by gastrointestinal nematodes. Animals.

[6] Facciolongo A.M., De Marzo D., Ragni M., Lestingi A., Toteda F. (2015). Use of alternative protein sources for finishing lambs. 2. Effects on chemical and physical characteristics and fatty acid composition of meat. Prog. Nutr..

[7] Nagpal R., Shrivastava B., Kumar N., Dhewa T., Sahay H., Puniya A.K., Singh R., Kamra D.N. (2015). Microbial feed additives. Rumen Microbiology: From Evolution to Revolution.

[8] Bernal-Barragán H., Cerrillo-Soto M.A., García-Mazcorro J.F., Juárez-Reyes A.S., Salem A.Z.M., Salem A.F.Z.M. (2013). Antibiotics in animal nutrition. Nutritional Strategies of Animal Feed Additives.

[9] Maron D.F., Smith T.J.S., Nachman K.E. (2013). Restrictions on antimicrobial use in food animal production: An international regulatory and economic survey. Glob. Health.

[10] Mnasri T., Hérault J., Gauvry L., Loiseau C., Poisson L., Ergan F., Pencréac’H G. (2017). Lipase-catalyzed production of lysophospholipids. OCL Oilseeds Fats Crop. Lipids.

[11] Van Rensburg C.E.J., Joone G.K., O’Sullivan J.F., Anderson R. (1992). Antimicrobial activities of clofazimine and B669 are mediated by lysophospholipids. Antimicrob. Agents Chemother..

[12] Zhao P.Y., Li H.L., Hossain M.M., Kim I.H. (2015). Effect of emulsifier (lysophospholipids) on growth performance, nutrient digestibility and blood profile in weanling pigs. Anim. Feed Sci. Technol..

[13] Brautigan D.L., Li R., Kubicka E., Turner S.D., Garcia J.S., Weintraut M.L., Wong E.A. (2017). Lysolecithin as feed additive enhances collagen expression and villus length in the jejunum of broiler chickens. Poult. Sci..

[14] Zampiga M., Meluzzi A., Sirri F. (2016). Effect of dietary supplementation of lysophospholipids on productive performance, nutrient digestibility and carcass quality traits of broiler chickens. Ital. J. Anim. Sci..

[15] Zhao P.Y., Kim I.H. (2017). Effect of diets with different energy and lysophospholipids levels on performance, nutrient metabolism, and body composition in broilers. Poult. Sci..

[16] Zhao P.Y., Zhang Z.F., Lan R.X., Liu W.C., Kim I.H. (2017). Effect of lysophospholipids in diets differing in fat contents on growth performance, nutrient digestibility, milk composition and litter performance of lactating sows. Animal.

[17] Wang Q.Q., Long S.F., Hu J.X., Li M., Pan L., Piao X.S. (2019). Effects of dietary lysophospholipid complex supplementation on lactation performance, and nutrient digestibility in lactating sows. Anim. Feed Sci. Technol..

[18] Rico D.E., Ying Y., Harvatine K.J. (2017). Short communication: Effects of lysolecithin on milk fat synthesis and milk fatty acid profile of cows fed diets differing in fiber and unsaturated fatty acid concentration. J. Dairy Sci..

[19] Lee C., Morris D.L., Copelin J.E., Hettick J.M., Kwon I.H. (2019). Effects of lysophospholipids on short-term production, nitrogen utilization, and rumen fermentation and bacterial population in lactating dairy cows. J. Dairy Sci..

[20] Sontakke U.B., Kaur H., Tyagi A.K., Kumar M., Hossain S.A. (2014). Effect of feeding rice bran lyso-phospholipids and rumen protected fat on feed intake, nutrient utilization and milk yield in crossbred cows. Indian J. Anim. Sci..

[21] Song W.-S., Yang J., Hwang I.H., Cho S., Choi N.-J. (2015). Effect of dietary lysophospholipid (LIPIDOL^TM^) supplementation on the improvement of forage usage and growth performance in Hanwoo heifer. J. Korean Soc. Grassl. Forage Sci..

[22] Kamande G.M., Baah J., Cheng K.J., McAllister T.A., Shelford J.A. (2000). Effects of tween 60 and tween 80 on protease activity, thiol group reactivity, protein adsorption, and cellulose degradation by rumen microbial enzymes. J. Dairy Sci..

[23] Ma N. (2004). Introduction of breeding stock and protection and utilization of genetic resources of sheep. J. Jilin Agric. Univ..

[24] Alton G.G., Jones L.M., Pietz D.E. (1975). Laboratory Techniques in Brucellosis.

[25] National Research Council (2007). Nutrient Requirements of Small Ruminants: Sheep, Goats, Cervids and New World Camelids.

[26] Liang J., Zhu L., Xu Z. (2006). Study on the determination of NH_4_^+^-N content in microbial fermentation liquor by indophenol blue spectrophotometric method. Food Ferment. Ind..

[27] Feng Z., Gao M. (2010). A modified spectrophotometric method for the determination of ammonia concentration in ruminal liquor. Anim. Husb. Feed Sci..

[28] Magoč T., Salzberg S.L. (2011). FLASH: Fast length adjustment of short reads to improve genome assemblies. Bioinformatics.

[29] Edgar R.C. (2013). UPARSE: Highly accurate OTU sequences from microbial amplicon reads. Nat. Methods.

[30] Henderson G., Yilmaz P., Kumar S., Forster R.J., Kelly W.J., Leahy S.C., Guan L.L., Janssen P.H. (2019). Improved taxonomic assignment of rumen bacterial 16S rRNA sequences using a revised SILVA taxonomic framework. PeerJ.

[31] Fan K., Yi Y., Liu Y., Li M., Wang J., Wang Z., Zhang H., Gu K. (2019). Preparation of soybean lysophospholipids and its biosafety analysis. China Oils Fats.

[32] Jenkins T.C., Gimenez T., Cross D.L. (1989). Influence of phospholipids on ruminal fermentation in vitro and on nutrient digestion and serum lipids in sheep. J. Anim. Sci..

[33] Russell J.B., Rychlik J.L. (2001). Factors that alter rumen microbial ecology. Science.

[34] Hwang I.H., Lee C.H., Kim S.W., Sung H.G., Lee S.Y., Lee S.S., Hong H., Kwak Y.C., Ha J.K. (2008). Effects of mixtures of Tween80 and cellulolytic enzymes on nutrient digestion and cellulolytic bacterial adhesion. Asian Australas. J. Anim. Sci..

[35] Rubino F., Carberry C., Waters S.M., Kenny D., Mccabe M.S., Creevey C.J. (2017). Divergent functional isoforms drive niche specialisation for nutrient acquisition and use in rumen microbiome. ISME J..

[36] Zhong S., Ding Y., Wang Y., Zhou G., Guo H., Chen Y., Yang Y. (2019). Temperature and humidity index (THI)-induced rumen bacterial community changes in goats. Appl. Microbiol. Biotechnol..

[37] Privé F., Newbold C.J., Kaderbhai N.N., Girdwood S.G., Golyshina O.V., Golyshin P.N., Scollan N.D., Huws S.A. (2015). Isolation and characterization of novel lipases/esterases from a bovine rumen metagenome. Appl. Microbiol. Biotechnol..

[38] Harfoot C.G., Hazlewood G.P., Hobson P.N., Stewart C.S. (1997). Lipid metabolism in the rumen. The Rumen Microbial Ecosystem.

[39] Caporaso J.G., Kuczynski J., Stombaugh J., Bittinger K., Bushman F.D., Costello E.K., Fierer N., Peña A.G., Goodrich J.K., Gordon J.I. (2010). QIIME allows analysis of high-throughput community sequencing data. Nat. Methods.

[40] Philippot L., Spor A., Hénault C., Bru D., Bizouard F., Jones C.M., Sarr A., Maron P.A. (2013). Loss in microbial diversity affects nitrogen cycling in soil. ISME J..

[41] Price M.N., Dehal P.S., Arkin A.P. (2010). FastTree 2—Approximately maximum-likelihood trees for large alignments. PLoS ONE.

