# Supplementary figures and images for: Dietary Supplementation of Lysophospholipids Affects Feed Digestion in Lambs
Source: Animals (Basel). 2019 Oct 15;9(10):805. doi: 10.3390/ani9100805 (PMC6826496; doi:10.3390/ani9100805)

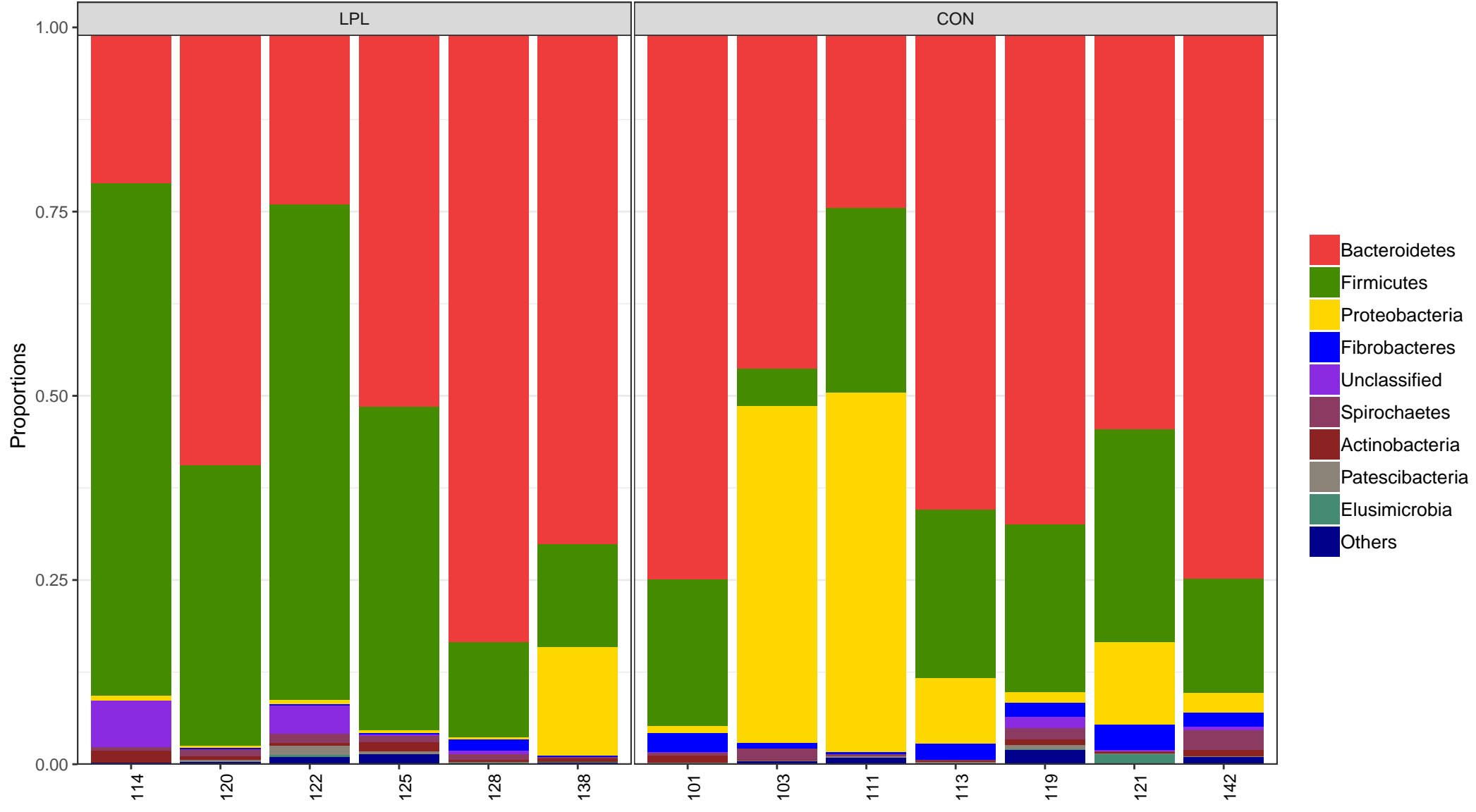

Supplement: Supplementary file 1 [file animals-09-00805-s001.zip › Figure S1 bar.ALL.phylum.xls.pdf]

CON LPL

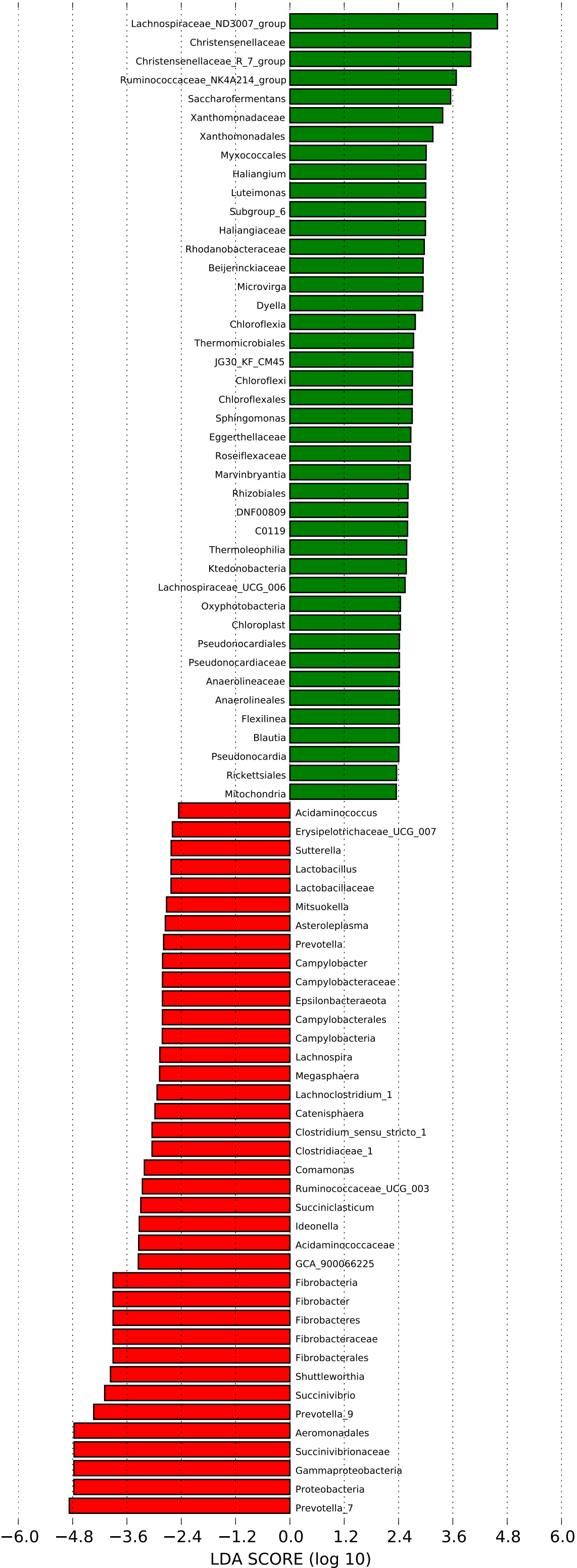

Supplement: Supplementary file 1 [file animals-09-00805-s001.zip › Figure S2 lefse_LDA.pdf]
